# Using automated texture features to determine the probability for masking of a tumor on mammography, but not ultrasound

**DOI:** 10.1186/s40001-017-0270-0

**Published:** 2017-08-30

**Authors:** Lothar Häberle, Carolin C. Hack, Katharina Heusinger, Florian Wagner, Sebastian M. Jud, Michael Uder, Matthias W. Beckmann, Rüdiger Schulz-Wendtland, Thomas Wittenberg, Peter A. Fasching

**Affiliations:** 1 University Breast Center for Franconia, Department of Gynecology and Obstetrics, Erlangen University Hospital, Friedrich Alexander University of Erlangen-Nuremberg, Comprehensive Cancer Center Erlangen-EMN, Erlangen, Germany; 2 Biostatistics Unit, Department of Gynecology and Obstetrics, Erlangen University Hospital, Erlangen, Germany; 3 Fraunhofer Institute for Integrated Circuits IIS, Erlangen, Germany; 4 University Breast Center for Franconia, Institute of Radiology, Comprehensive Cancer Center EMN, Erlangen University Hospital, Friedrich Alexander University of Erlangen-Nuremberg, Erlangen, Germany; 5 Division Hematology/Oncology, Department of Medicine, David Geffen School of Medicine, University of California at Los Angeles, Los Angeles, CA USA

**Keywords:** Mammography screening, Texture analysis, Masking, Mammographic density, Sensitivity, Risk prediction, Variable selection

## Abstract

**Background:**

Tumors in radiologically dense breast were overlooked on mammograms more often than tumors in low-density breasts. A fast reproducible and automated method of assessing percentage mammographic density (PMD) would be desirable to support decisions whether ultrasonography should be provided for women in addition to mammography in diagnostic mammography units. PMD assessment has still not been included in clinical routine work, as there are issues of interobserver variability and the procedure is quite time consuming. This study investigated whether fully automatically generated texture features of mammograms can replace time-consuming semi-automatic PMD assessment to predict a patient’s risk of having an invasive breast tumor that is visible on ultrasound but masked on mammography (mammography failure).

**Methods:**

This observational study included 1334 women with invasive breast cancer treated at a hospital-based diagnostic mammography unit. Ultrasound was available for the entire cohort as part of routine diagnosis. Computer-based threshold PMD assessments (“observed PMD”) were carried out and 363 texture features were obtained from each mammogram. Several variable selection and regression techniques (univariate selection, lasso, boosting, random forest) were applied to predict PMD from the texture features. The predicted PMD values were each used as new predictor for masking in logistic regression models together with clinical predictors. These four logistic regression models with predicted PMD were compared among themselves and with a logistic regression model with observed PMD. The most accurate masking prediction was determined by cross-validation.

**Results:**

About 120 of the 363 texture features were selected for predicting PMD. Density predictions with boosting were the best substitute for observed PMD to predict masking. Overall, the corresponding logistic regression model performed better (cross-validated AUC, 0.747) than one without mammographic density (0.734), but less well than the one with the observed PMD (0.753). However, in patients with an assigned mammography failure risk >10%, covering about half of all masked tumors, the boosting-based model performed at least as accurately as the original PMD model.

**Conclusion:**

Automatically generated texture features can replace semi-automatically determined PMD in a prediction model for mammography failure, such that more than 50% of masked tumors could be discovered.

## Background

The effort to improve breast cancer detection faces several challenges. One of these is how to integrate different diagnostic methods into a single diagnostic process. Although mammography screening programs do not include ultrasonography, some diagnostic mammography units do use ultrasound. However, no systematic guidelines are currently available to indicate when ultrasound should be used and when not. Some diagnostic units use ultrasound for every patient, but others do so only for certain indications, such as dense breasts, or if the patient requests it [1]. The reasons for the unsystematic way in which ultrasound is used lie in the associated costs and the lack of prediction models capable of identifying those patients in whom an additional method would increase sensitivity without necessarily decreasing specificity.

A recent study investigated risk factors for masking of invasive breast tumors on mammograms [2]. The authors showed that the probability of a tumor being detected on ultrasound but not on mammography (mammography failure) depended on the patient’s age, body mass index (BMI), previous breast surgery, and percentage mammographic density (PMD). PMD was the strongest predictor of mammography failure. Tumors in dense breasts were overlooked more often than tumors in low-density breasts. Other studies, in which ultrasound was incorporated into screening programs for women with dense breasts, have also reported that sensitivity for tumor detection increased but specificity decreased when ultrasound was added to mammography [3, 4].

In clinical practice, PMD assessment has still not been included in clinical routine work, as there are issues of interobserver and intermethod variability and the procedure is quite time consuming [5, 6]. In research settings, two readers usually determine the proportion of dense breast using semiquantitative software analysis. In clinical routine, a fast, reproducible and automated method of assessing PMD would be desirable to help physicians decide whether ultrasonography should be provided for a woman in addition to mammography. Since texture features in the mammogram are useful for predicting the risk of breast cancer and estimating mammographic density [7–16], applying an adequate feature set might be a way of obtaining information from the mammogram that would be helpful in replacing mammographic density assessment.

The aim of the present study was, therefore, to investigate to which extent fully automatically generated texture features can replace time-consuming semi-automatic assessment of mammographic density to predict a patient’s risk of having an invasive breast tumor that is visible on ultrasound but not on mammography, in a diagnostic mammography setting.

## Methods

### Study population

The patients in this retrospective study of prospectively acquired data were selected from all breast cancer patients who were diagnosed and treated at the University Breast Center for Franconia, Erlangen University Hospital, between 2000 and 2009 and whose initial mammography was performed there, i.e., all mammograms were done at the point of the initial diagnosis of breast cancer. Patients are referred to the breast center to identify the need for a diagnostic biopsy. No invasive procedures had been carried out before the patient’s referral to the hospital, and women whose breast cancer was initially discovered in the screening program were not included. The institution’s diagnostic procedures require that all patients are examined with both mammography and additional ultrasound, regardless of the result of either imaging method and regardless of any patient characteristics.

Patients were selected in the following hierarchical order from a total of 3974 breast cancers registered in the breast center’s database: invasive breast cancer (excluding 486 patients with in situ cancers); no contralateral breast cancer (excluding 412 patients); mammography at primary diagnosis performed at the university breast center (excluding 1688 patients); physical availability of mammograms for the affected and contralateral sides (excluding five patients); availability of a structured Breast Imaging Reporting and Data System (BI-RADS) or analogous assessment of the mammogram and ultrasound scan (excluding 49 patients).

### Clinical data

All patient characteristics were documented as part of the certification processes required by the German Cancer Society (*Deutsche Krebsgesellschaft*) and by the German Society for Breast Diseases (*Deutsche Gesellschaft für Senologie*) [17].

Mammograms for the breast cancer patients participating were considered as mammography failures and as masked if the diagnostic assessment of the mammogram was BI-RADS 2 or 1. A total of 108 unsuspicious mammograms from patients with suspicious lesions on the corresponding ultrasound were reviewed again, and one case was found that was reclassified as BI-RADS 4 and no longer regarded as a mammography failure.

### Observed mammographic density

The mammograms were digitized using the CAD PRO Advantage^®^ film digitizer (VIDAR Systems Corporation, Herndon, Virginia, USA). Both analog images and printouts of digital mammograms were used. Quantitative computer-based threshold density assessments were carried out in 2011 and 2012 by two different readers (C.C.H., K.H) with 6 and 5 years of experience in the method used [18]. Each mammogram was read in random order by both readers independent of each other. To assess the density proportion, the readers used the Madena Software Program, version X (Eye Physics, LLC, Los Alamitos, CA, USA). Only the measurements for the contralateral healthy breast were used for analysis. Both readers were unaware of any previous classifications or pathological findings. Averages of the two observers’ values for PMD were used for analysis.

### Image analysis

A total of 363 texture features were calculated to characterize the mammographic images in the present study. Since an image is made up of pixels, it can be represented as a matrix in which each entry is an integer from 0 to 255, describing the gray value of the corresponding pixel. Generally speaking, texture features provide information about the gray-level distribution within an image or image region to distinguish between light and dark images—in this case, dense and soft breasts. Texture features may also provide information about the spatial relationship between gray levels, to distinguish between homogeneous and heterogeneous images and between cloudy and sharp patterns. There are also features that recognize periodicity of pattern [9].

Families of texture features used for analyses have been described previously [7]. Briefly:

*Moment*-*based features* (*n* = 76 features) They describe the gray-level distribution without regard to the spatial relationships of pixels. The central moments (mean, variance, skewness, kurtosis), normalized central moments (NCM), and transformations of the NCM belong to this feature family.

*Histogram features* (*n* = 16) The full spectrum of all gray levels was equally divided into 16 categories. The frequency of pixels in a specific category is called the histogram feature. Obviously, there are 16 histogram features.

*Markovian features* (*n* = 93) They describe the spatial relationship of pixels. They are computed on the basis of measurements derived from co-occurrence matrices or sum and difference histograms. A co-occurrence matrix measures the probability that two pixels of certain gray levels will be positioned at a particular distance and orientation. A sum histogram and accordingly a difference histogram count all combinations of two pixels with a particular distance, orientation and sum and difference of gray levels, respectively.

*Regional features* (*n* = 48) Pixels are clustered to regions in accordance with a similarity criterion. The criterion may depend on the distance, or the gray level, or both. A regional feature then characterizes the number of regions, the shape of the regions, or the gray-level distribution of the regions.

*Run*-*length features* (*n* = 60) They examine runs of similar gray levels in an image. Runs may be labeled according to their length, gray value, and direction. Long runs of the same gray value correspond to coarser textures, whereas shorter runs correspond to finer textures.

*Fourier features* (*n* = 33) They characterize image regions that show periodic structures. The image was transformed to a Fourier space. Then features are extracted from different portions of the Fourier space corresponding to low- and high-frequency image content.

*Wavelet features* (*n* = 37) They characterize spectral properties such as periodic structures at various spatial resolution levels. The image was iteratively transformed into four sub-images based on frequency content and orientation using wavelets. The features describe the energy of the sub-images. Sub-images of different levels correspond to different scales. Hence, this feature group extracts features for different scales.

### Statistical analysis: preselection of texture features

Box plots were created for all 363 features. Four very skew-distributed features were excluded after visual inspection of the box plots. The features were randomly ordered, and Spearman’s correlation coefficients were calculated for all pairs of features among the remaining 359 features. Each feature with a correlation >0.98 with a higher ranked feature was excluded to obtain a feature set without highly correlated features. Some basic features (central moments, histogram features) that had proved to be predictive in a previous study [7] were accepted without preselection. In total, 218 features were considered for further analysis.

### Statistical analysis: prediction of PMD

Identifying relevant predictors for PMD among the relatively high number of texture features was a challenge, which can be summed up as follows. The complete dataset was randomly divided into two parts: one training set with about two-thirds of the patients and one validation set with about one-third of the patients. Different feature selection methods and regression techniques, respectively, were applied to training data to obtain PMD predictions. All of the regression techniques considered comprise a bundle of candidate models characterized by a tuning parameter *λ*. The optimal *λ* has to be determined before a specific prediction model representing the regression technique can be fitted to predict PMD. The following regression techniques were applied to training data:

*Univariate selection* For each feature, a linear regression model with the specific feature was set up and a global F test was performed. The features were ordered according to increasing *p* values for these F tests. The *λ* top-ranked features were selected and included in a multiple linear regression model. Here *λ*, ranging from 1 to 150, is a tuning parameter representing the number of selected features.

*Lasso* (least absolute shrinkage and selection operator) [19] It is a regression technique in which the regression coefficients are shrunk towards zero. The amount of shrinkage is controlled by a tuning parameter *λ*. Depending on the value of *λ*, a number of coefficients reach exactly zero, which means that lasso is also a variable selection method. In this study, we set up a regression model with all features. The coefficients of the features were shrunk by variation of *λ*. In contrast to the usual regression models, lasso can deal with large numbers of predictors.

*Component*-*wise gradient boosting* [20, 21] It fits a regression model iteratively. It starts with an empty model without any predictors. In each iteration, the best-performing predictor is added to the model with a small step size, or its coefficient is updated if it was included before. More relevant predictors are included earlier than less relevant ones. The number of iterations *λ* is a tuning parameter that controls the number of selected predictors and the shrinkage of the coefficients.

*Random forest* [22] A forest consisting of many decision trees was fitted to the data. Each tree is based on binary splits of randomly chosen features. This technique already takes into account overfitting during the fitting process, and nonlinear relationships between predictors and outcome are considered. The number of variables randomly sampled as candidates at each split was controlled by a tuning parameter *λ*.

The optimal *λ* for each method except for random forest was found by 10-fold cross-validation on the training dataset. For a given value of *λ*, the prediction model was estimated on nine folds and then applied on the tenth fold. The mean squared error (MSE) was taken as the evaluation measure. The MSE is a summary measure of the differences between the observed PMD values for patients in the tenth fold, which was not used for model building, and their predicted PMD values using the regression model. This procedure was done ten times, leaving one fold out at a time, and the average MSE was calculated. The *λ* value with the smallest average MSE was regarded as the optimal *λ*. The whole training set was finally used to fit a regression model with the optimal *λ*. At random forest, various forests depending on *λ* were fitted to the training dataset, and the forest with the smallest out-of-bag error was selected.

The procedures described above resulted in four regression models each for predicting PMD. Four continuous variables with PMD predictions were generated on training data and validation data, respectively, by applying the regression models to the corresponding datasets.

### Statistical analysis: prediction of masking

The binary outcome variable “masking status” was created to distinguish between patients whose tumor was detected with ultrasonography but not with mammography (status = 1) and those whose tumor was detected with mammography, regardless of the ultrasonography result (status = 0). The primary aim of the study was to generate a continuous variable that predicts PMD from texture features (“predicted PMD”) and could replace the semi-automatically determined predictor PMD (“observed PMD”) in the prediction model for masking proposed in a previous study [2].

The new PMD predictors based on univariate selection, lasso, boosting and random forest, respectively, were each entered into a logistic regression model on the training data, together with the clinical predictors from the previously proposed prediction model for masking, i.e., age (continuous), BMI (continuous), previous breast surgery (yes/no), HRT status and menopausal status (premenopausal, postmenopausal and no HRT usage, postmenopausal and HRT usage), and imaging technique (digital/analog) [2].

The logistic regression models were evaluated on the validation dataset to measure their performance in new patients. They were fitted on the training dataset and, again, the MSE on the validation dataset was taken as a performance criterion. Here, the MSE is a summary statistic of the differences between the observed masking status (either 0 or 1) of patients from the validation set and the expected probability obtained from the model (between 0 and 1) for these patients having status = 1. Furthermore, a null model without any predictors, the clinical logistic regression model without PMD, and a logistic regression model with clinical predictors and the observed PMD as in [2] were fitted on the training data and their MSEs were calculated with the validation data.

The predictive performance of the logistic regression models, in terms of discriminating between overlooked and detected tumors, was assessed using the receiver operating characteristic (ROC) curve, the area under the ROC curve (AUC) and the continuous net reclassification improvement (NRI). Roughly speaking, the continuous NRI is the proportion of patients with overlooked or detected tumors who are correctly given a higher or lower predicted probability of masking by the regression model with mammographic density, rather than by the clinical model without PMD, corrected by wrongly assigned lower or higher probabilities [23].

To demonstrate a possible future application of a prediction model, various cut-off points for the masking risk between 0 and 100% were defined, e.g., 12%. Subjects were classified as “low risk” if the prediction model assigned a masking risk below 12%. Otherwise, they were classified as “high risk.” Discovery rates—i.e., the proportion of patients classified as “high risk” among true masked tumors—are presented.

To overcome the drawbacks of only splitting the data into training and validation sets once, we divided the dataset several times into training and validation sets and repeated the procedure described above each time [24]. More precisely, 3-fold cross-validation with 100 repetitions was done. For each regression technique for predicting PMD, the average value of the 300 MSEs of the corresponding logistic regression models was taken as a final evaluation criterion. The regression technique with the smallest average MSE in logistic regression is regarded as the best method (the “winner” method) for substituting the semi-automatically assessed PMD by an automatically generated PMD in a logistic regression model for predicting masking. The average AUC and average NRI were used as further criteria.

The best prediction method was applied to the whole dataset to obtain the final prediction model for masking. This was done by repeating all model building steps, this time not on the training data, but on the complete dataset. That is, the tuning parameter *λ* was determined as described above and a corresponding regression model was fitted on the complete dataset to obtain predicted PMD values, which were entered into a logistic regression together with clinical predictors.

### Statistical analysis: prediction of PMD (part 2)

The best regression technique for substituting the observed PMD to predict masking as well as possible does not need to be the most accurate technique for predicting PMD itself. A comparison of the regression techniques in relation to PMD prediction performance was a secondary study aim. The prediction performance of the regression models was assessed using the average MSE and the average *R* ^2^ statistic on validation datasets.

Calculations were carried out using the R system for statistical computing (version 3.0.1; R Core Team, Vienna, Austria, 2013). Particularly, the R packages mboost (version 2.2-3), randomForest (version 4.6-7) and glmnet (version 1.9-5) were used to fit boosting, random forest and lasso models.

## Results

### Patient characteristics

A total of 1334 patients were included in the analysis. The percentages of missing data for each variable were below 5%. Missing values were imputed, as described previously in [2]. In all, 107 patients (8.0%) had tumors that were detected with ultrasound alone but not with mammography. Clinical data are shown in Table 1.

**Table 1:** Patient characteristics in relation to mammography failure (yes/no)

Characteristic	Visible on mammography and US		Visible only on US (mammography failure)	
	Mean or *n*	SD or %	Mean or *n*	SD or %
Age	60.2	12.5	52.5	12.1
BMI	26.4	4.7	23.7	3.5
PMD	34.5	18.3	51.3	20.5
Previous breast surgery				
No	1080	87.4	79	80.6
Yes	156	12.6	19	19.4
Menopausal and HRT status				
Premenopausal	269	21.8	46	46.9
Postmenopausal and no HRT	721	58.3	28	28.6
Postmenopausal and HRT	246	19.9	24	24.5
Imaging technique				
Analog	761	61.6	55	56.1
Digital	475	38.4	43	43.9

Mean and standard deviation (SD) are shown for continuous characteristics, and frequency and percentage for categorical characteristics

*BMI* body mass index,* HRT* hormone replacement therapy,* PMD* percentage mammographic density,* US* ultrasonography

### Prediction of PMD (secondary study aim)

**Fig. 1:**
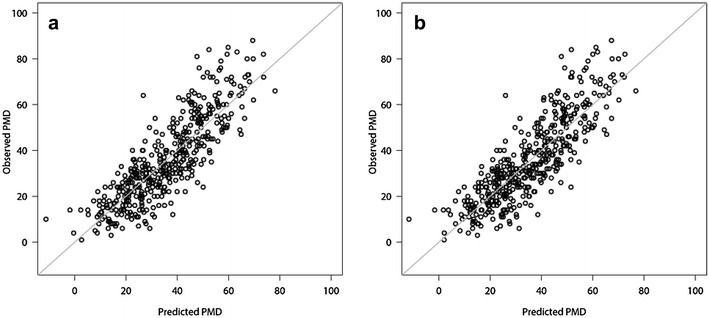
Predicted and observed percentage mammographic density (PMD) values on a validation dataset (one-third of the patients), based on linear regression models fitted on a training dataset (two-thirds of the patients) using lasso (**a**) and boosting (**b**)

The results, after the evaluation procedures were applied to each of the four prediction methods, are summarized in Table 2. Lasso turned out to be the most accurate feature selection method and had a slightly smaller cross-validated prediction error MSE than boosting. Univariate selection and random forest performed distinctly less well than lasso and boosting. As expected, smaller prediction errors are reflected in larger *R* ^2^ values. The average number of selected features is relatively large, with more than half of all considered features. Fig. 1 shows the observed mammographic density and predictions on a validation dataset using lasso and boosting models that had previously been fitted on training data.

**Table 2:** Prediction of PMD

Method	MSE	*R* ^2^	*N*
Univariate selection	117.0 (8.6)	0.67 (0.02)	132.5 (9.7)
Lasso	111.9 (8.4)	0.69 (0.02)	108.8 (12.9)
Boosting	113.0 (8.6)	0.68 (0.02)	126.1 (8.8)
Random forest	120.2 (9.7)	0.66 (0.03)	–^a^

Summary statistics (mean and standard deviation) of mean squared error (MSE) and *R* ^2^ obtained from (linear) regression models with selected features, as well as the number of selected features N, are shown. All measurements were obtained by 3-fold cross-validation with 100 repetitions

*MSE* mean squared error,* PMD* percentage mammographic density

^a^There was no variable selection with random forest

After lasso and boosting had been found to be the best prediction techniques, a lasso and a boosting model were fitted on the whole dataset for analysis in greater detail (Table 3). Features from all feature families were selected. Nearly all histogram features were selected by both lasso and boosting. A higher than average number of features were taken from the wavelet and regional family. Nearly 90% of the boosting features were also selected by lasso. As expected, features that strongly correlated with PMD were preferred in the selection procedures. The features with the highest correlation with PMD within a feature family were almost always chosen in both models. The median correlation coefficients of the selected features were similar to the median correlation coefficients of the complete feature set, indicating that in total, selected features and not-selected features behaved similarly with regard to correlation with PMD. Particularly, many features that hardly correlate with PMD were selected.

**Table 3:** Selected texture features for predicting percentage mammographic density (PMD)

Feature family	Number of features				Correlation with PMD Median (min., max.)^c^		
	All	Lasso^a^	Boosting^a^	Common^b^	All	Lasso	Boosting
Fourier	12	9	9	6	0.16 (0.03, 0.28)	0.10 (0.03, 0.28)	0.12 (0.03, 0.28)
Histogram	14	13	13	12	0.18 (0.00, 0.25)	0.19 (0.00, 0.25)	0.17 (0.00, 0.25)
Markovian	37	24	24	20	0.44 (0.00, 0.72)	0.39 (0.00, 0.72)	0.43 (0.00, 0.72)
Moment-based	70	54	33	32	0.21 (0.00, 0.61)	0.21 (0.00, 0.61)	0.21 (0.00, 0.61)
Regional	45	36	32	28	0.22 (0.01, 0.52)	0.23 (0.03, 0.52)	0.20 (0.01, 0.52)
Run length	28	18	15	12	0.59 (0.04, 0.71)	0.60 (0.15, 0.70)	0.62 (0.04, 0.71)
Wavelet	12	10	8	8	0.24 (0.01, 0.42)	0.26 (0.06, 0.42)	0.26 (0.06, 0.31)
Total	218	164	134	118			

^a^Selected number of features using lasso and boosting method, respectively, to predict PMD. Prediction models were fitted on the complete dataset. The tuning parameters were estimated by cross-validation

^b^Number of features selected both by lasso and boosting

^c^Each feature was correlated with PMD. Summary statistics (median, minimum, maximum) of Spearman correlation coefficients between (all and selected) features and PMD are shown

### Prediction of masking (primary study aim)

The PMD prediction from boosting (cross-validated MSE, 0.0654) and, slightly less well, lasso (0.0655) turned out to be the best replacement for the observed PMD in the logistic regression model for predicting masking (Table 4). The original logistic regression model with observed PMD, however, was more accurate (0.0645). Each model with observed or predicted PMD performed better than the clinical model without PMD (0.0657).

**Table 4:** Prediction of masking

Method	MSE	AUC	NRI	Reclassification	
				Correctly upwards	Correctly downwards
Null^a^	0.0682 (0.0095)	0.500 (0.000)			
Clinical findings^b^	0.0657 (0.0085)	0.734 (0.037)			
Univariate selection^c^	0.0656 (0.0085)	0.743 (0.036)	27.9 (16.2)	57.9 (9.1)	56.1 (3.0)
Lasso^c^	0.0655 (0.0084)	0.747 (0.036)	33.1 (15.6)	60.0 (8.7)	56.6 (3.0)
Boosting^c^	0.0654 (0.0084)	0.747 (0.036)	32.5 (15.5)	59.8 (8.6)	56.5 (3.0)
Random forest^c^	0.0656 (0.0087)	0.739 (0.035)	4.4 (16.3)	45.1 (9.0)	57.1 (3.4)
Observed PMD^d^	0.0645 (0.0082)	0.753 (0.036)	35.7 (14.4)	58.5 (8.2)	59.4 (2.9)

Summary statistics (mean and standard deviation) of MSE, AUC, and the net reclassification improvement (NRI) in percentages obtained from logistic regression models with clinical predictors and the observed or predicted PMD using various regression methods. All measurements were obtained by 3-fold cross-validation with 100 repetitions

*AUC* area under the curve,* BMI* body mass index,* HRT* hormone replacement therapy,* MSE* mean squared error,* NRI* net reclassification improvement,* PMD* percentage mammographic density

^a^Logistic regression model without any predictors

^b^Logistic regression model with clinical predictors (age, BMI, prior breast surgery, menopausal and HRT status, imaging technique) but without PMD

^c^Logistic regression model with clinical predictors and PMD predicted from texture features using univariate selection, lasso, boosting, or random forest

^d^Logistic regression model with clinical predictors and the original PMD values (“observed PMD”)

The AUC values of the logistic regression models with predicted PMD based on lasso and boosting (cross-validated AUC, both 0.747) were in the middle between that of the clinical model (0.734) and that of the observed PMD model (0.753), indicating an improved ability of these models to differentiate between patients whose tumor will be overlooked and patients whose tumor will not be overlooked in comparison with the clinical model. As with the MSE, the AUCs for univariate selection and random forest were poorer than those of boosting and lasso, but still better than that of the clinical model without PMD.

All methods except random forest correctly increased the predicted probabilities of masking for the majority of patients with a masked mammogram in comparison with the clinical model (“correct reclassification upwards” in Table 4). Lasso and boosting showed the largest improvement, followed by the model with the observed PMD and univariate selection. In patients without a masked mammogram, all methods correctly decreased the predicted probabilities for the majority of patients (“correct reclassification downwards” in Table 4). In total, the reclassification improvement of the model with the observed PMD (cross-validated NRI, 35.7%) was slightly better than the models with predicted PMD based on boosting (32.5%) or lasso (33.1%), and much better than the models with predicted PMD using univariate selection (27.9%) or random forest (4.4%, Table 4).

**Fig. 2:**
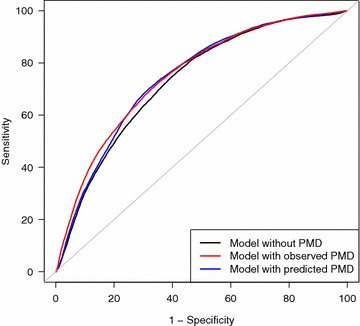
Cross-validated receiver operating characteristic (ROC) curves, showing the discriminative value of logistic regression models, each with clinical predictors but with different percentage mammographic density (PMD) measures (without PMD, with observed PMD, and with predicted PMD using boosting)

Discovery rates are presented for the boosting model, the winner in the method comparison, in Table 5, and compared with the clinical model and the observed PMD model. The discovery rates for the boosting model are generally better than those of the clinical model. They are slightly better than those for the observed PMD model for cut-off points up to 10%, but poorer thereafter. For instance, if a physician decides to offer ultrasound to women with a predicted risk of masking of more than 10%, then 57.7% of all tumors that are missed with diagnosis relying on mammography alone will be detected with the boosting model, in comparison with 55.6% with the original PMD model. Assuming that the general population has a similar risk distribution, additional ultrasound would be necessary in 26.4% of all women presenting at a diagnostic mammography unit. The ROC curves shown in Fig. 2 for all possible cut-off points confirm that the boosting model lies between the clinical model and the observed PMD model. Table 6 lists the coefficients of the logistic regression model with predicted PMD using boosting.

**Table 5:** Discovery rates for three models and different cut-off points

Cut-off point for predicted masking risk (%)^a^	Frequency above cut-off point (%)^b^	Discovery rates for tumors not seen on mammography (%)		
		Clinical model^c^	Boosting PMD model^d^	Observed PMD model^e^
5	47.5	81.8	80.9	78.9
10	26.4	54.5	57.7	55.6
12	20.0	44.8	47.4	48.7
15	13.6	32.9	35.1	39.7
20	7.5	16.2	21.0	25.4

All measurements were obtained by 3-fold cross-validation with 100 repetitions

*BMI* body mass index,* HRT* hormone replacement therapy,* PMD* percentage mammographic density

^a^Patients were classified into a “high-risk” group if the prediction model assigned a masking risk above the cut-off point. Discovery rates are defined as the proportion of masked tumors in the “high-risk” group

^b^Proportion of “high risk” classified patients in the total study population, using boosting-based prediction model

^c^Logistic regression model with the clinical predictors age, BMI, previous breast surgery, menopausal and HRT status, and imaging technique

^d^Logistic regression model with the same clinical predictors and additionally PMD predicted by a boosting regression model beforehand

^e^Logistic regression model with the clinical predictors and the observed PMD

**Table 6:** Logistic regression model for predicting masking with predicted PMD based on boosting

Variable	Coefficient (standard error)
Baseline	–0.906 (1.308)
Age (year)	–0.018 (0.014)
BMI (kg/m^2^)	–0.080 (0.033)
Previous breast surgery	
No^a^	0
Yes	0.502 (0.286)
Menopausal and HRT status	
Premenopausal^a^	0
Postmenopausal and no HRT	–0.530 (0.357)
Postmenopausal and HRT	0.208 (0.355)
Imaging technique	
Analog^a^	0
Digital	0.416 (0.223)
Predicted PMD	0.032 (0.009)

The model is fitted on the complete dataset. To estimate a patient’s risk for masking, the following steps are necessary: texture features values are calculated from the mammogram, the boosting regression model is applied to obtain the predicted PMD, and patient characteristics and predicted PMD are linearly combined with the logistic regression coefficient to obtain interim value *z.* Finally, exp (*z*)/(1 + exp (*z*)) is the predicted risk for masking

^a^Reference category

## Discussion

The study shows that prediction of masking on diagnostic mammograms can be improved if mammographic density estimations using texture features are added to a prediction rule based on age, BMI, prior surgery, menopausal and HRT status, and imaging technique. However, the overall performance of such a prediction model was inferior to a prediction model with semi-automated measurements of PMD. Nonetheless, a clinically relevant group of patients was identified in which the new prediction model performed at least as well as the traditional one. A clinical application for this automated algorithm might be envisaged in automated fusion machines performing mammography and additionally ultrasound in case of increased risk of masking.

In patients with a predicted risk of masking greater than 10%, the boosting model outperformed the semi-automated prediction model from [2] in relation to the discovery rate of masked tumors. Lowering the cut-off point would lead to similar performances with both models. Furthermore, the discovery rate would increase, but the proportion of patients to whom ultrasound should be offered would also increase. Using higher cut-off points would reduce the number of patients requiring additional ultrasound but only a minority of all tumors not seen on mammography would be discovered. It appears, therefore, that with a discovery rate that was desirably high for clinical purposes (e.g., >50% when taking a 10% risk as the cut-off point), boosting-based mammographic density estimations might be able to replace semi-automated assessment of mammographic density without any loss of accuracy. This procedure could be implemented after further empirical validation.

Incorporating additional imaging methods into a diagnostic algorithm always harbors a risk of further invasive interventions being carried out in women who do not have a malignant lesion. It is, therefore, important to ensure that the cohort of women for whom a recommendation for additional diagnostic procedures is being developed is characterized very carefully. For example, women with high breast density values are offered ultrasound in addition to mammography in more than 24 states in the US [25]. In screening programs, it has been shown that breast density should not be the only criterion for whether additional diagnostic workup is justified, since not all women with a high mammographic density are at high risk for the occurrence of interval cancers, and other predictors also influence the risk of an interval cancer [26]. Similarly, the accuracy for predicting masking could be improved using additional oogenetic factors that were not taken into account in the present study and possibly genetic factors as well. Increasing the accuracy might reduce the number of unnecessary invasive interventions.

The texture feature selection process was carried out following a prespecified plan. Univariate selection is a simple method that does not take correlations among features into account. It is known to perform less well in general than more sophisticated methods such as lasso [24], a result that was confirmed in this study and recently in [27]. Lasso and boosting performed similarly, although the model fitting is rather different. However, the two methods share the common feature that variable selection is a continuous process that leads to “weakly” selected features in addition to strong predictors. All regression techniques except for random forest treated features as linear predictors that were summed up in a certain way to estimate PMD. A further study might show whether nonlinear usage of the features at lasso and boosting would improve the prediction. Random forest can deal with nonlinear effects, but its performance was poorest. A promising strategy in medical image analysis is the use of deep learning algorithms, in particular convolutional neural networks [28]. In [15], unsupervised deep learning was applied to texture features from mammograms.

Double cross-validation with an inner loop to specify the prediction model and an outer loop to compute model performance measures was carried out to ensure that all model building steps were performed completely independent of the validation step [29, 30]. That is, all reported measures were based on data that were not used for model building. Otherwise, the measures would have been over-optimistic. Preselection of texture features was performed once on the complete dataset before the actual model building and model assessment procedures started, and was not repeated during later steps. It did not employ any information related to the outcome to avoid biasing model assessments. Schild et al.  [31] and Häberle et al.  [27] provide examples of double (cross-)validation being applied in gynecological studies. Another strength of this study is the use of a large cohort of more than 1000 breast cancer patients. The cohort did not focus on women with a high mammographic density, but included all women attending a diagnostic mammography unit, regardless of any criteria other than admission.

This study has certain limitations. The results are restricted to a clinical diagnostic setting in which the complementary use of breast ultrasound and mammography is already routine practice. No direct conclusions can be drawn with regard to application in a screening setting, nor can any conclusions regarding specificity be drawn at present. At most, the discovery rates described can serve as preliminary estimations for discovery rates in a screening setting. Further research in the screening setting is warranted to assess the specificity and feasibility of the algorithm.

## Conclusions

Automatically generated texture features can replace semi-automatically determined PMD values in a prediction model for a patient’s risk for having a masked tumor, such that more than 50% of masked tumors could be discovered. Automated risk prediction allows implementation of observer-independent, model-based risk calculation in high-throughput mammography settings. After further empirical validation, our risk prediction algorithm might be implemented in fusion machines performing mammography and additionally ultrasound if necessary. The sophisticated statistical procedures applied in this study follow a prespecified, systematic plan and are described generally enough to be easily adapted for other study purposes.

## Abbreviations

AUC: area under the ROC curve
BI-RADS: breast imaging reporting and data system
BMI: body mass index
HRT: hormone replacement therapy
NRI: net reclassification improvement
PMD: percentage mammographic density
ROC: receiver operating characteristic

## References

[1] Sharpe R, Levin D, Rao V, Parker L. Breast imaging utilization trends in the medicare population from 2005 to 2011. Conference: Radiological Society of North America 2013 Scientific Assembly and Annual Meeting. 2013.

[2] Häberle L, Fasching PA, Brehm B, Heusinger K, Jud SM, Loehberg CR, Hack CC, Bayer CM, Lux MP, Hartmann A, Vachon C, Meier-Meitinger M, Uder M, Beckmann MW, Schulz-Wendtland R (2016). Mammographic density is the main correlate of tumors detected on ultrasound but not on mammography. Int J Cancer.

[3] Berg WA, Blume JD, Cormack JB, Mendelson EB, Lehrer D, Bohm-Velez M, Pisano ED, Jong RA, Evans WP, Morton MJ, Mahoney MC, Larsen LH, Barr RG, Farria DM, Marques HS, Boparai K (2008). Combined screening with ultrasound and mammography vs mammography alone in women at elevated risk of breast cancer. JAMA.

[4] Kolb TM, Lichy J, Newhouse JH (2002). Comparison of the performance of screening mammography, physical examination, and breast US and evaluation of factors that influence them: an analysis of 27,825 patient evaluations. Radiology.

[5] Brandt KR, Scott CG, Ma L, Mahmoudzadeh AP, Jensen MR, Whaley DH, Wu FF, Malkov S, Hruska CB, Norman AD, Heine J, Shepherd J, Pankratz VS, Kerlikowske K, Vachon CM (2016). Comparison of clinical and automated breast density measurements: implications for risk prediction and supplemental screening. Radiology.

[6] Destounis S, Arieno A, Morgan R, Roberts C, Chan A (2017). Qualitative versus quantitative mammographic breast density assessment: applications for the US and Abroad. Diagnostics.

[7] Häberle L, Wagner F, Fasching PA, Jud SM, Heusinger K, Loehberg CR, Hein A, Bayer CM, Hack CC, Lux MP, Binder K, Elter M, Munzenmayer C, Schulz-Wendtland R, Meier-Meitinger M, Adamietz BR, Uder M, Beckmann MW, Wittenberg T (2012). Characterizing mammographic images by using generic texture features. Breast Cancer Res.

[8] Heine JJ, Carston MJ, Scott CG, Brandt KR, Wu FF, Pankratz VS, Sellers TA, Vachon CM (2008). An automated approach for estimation of breast density. Cancer Epidemiol, Biomark Prev.

[9] Manduca A, Carston MJ, Heine JJ, Scott CG, Pankratz VS, Brandt KR, Sellers TA, Vachon CM, Cerhan JR (2009). Texture features from mammographic images and risk of breast cancer. Cancer Epidemiol Biomark Prev.

[10] Heine JJ, Scott CG, Sellers TA, Brandt KR, Serie DJ, Wu FF, Morton MJ, Schueler BA, Couch FJ, Olson JE, Pankratz VS, Vachon CM (2012). A novel automated mammographic density measure and breast cancer risk. J Natl Cancer Inst.

[11] Olson JE, Sellers TA, Scott CG, Schueler BA, Brandt KR, Serie DJ, Jensen MR, Wu FF, Morton MJ, Heine JJ, Couch FJ, Pankratz VS, Vachon CM (2012). The influence of mammogram acquisition on the mammographic density and breast cancer association in the Mayo Mammography Health Study cohort. Breast Cancer Res.

[12] Fowler EE, Vachon CM, Scott CG, Sellers TA, Heine JJ (2014). Automated percentage of breast density measurements for full-field digital mammography applications. Acad Radiol.

[13] Gastounioti A, Conant EF, Kontos D (2016). Beyond breast density: a review on the advancing role of parenchymal textures analysis in breast cancer risk assessment. Breast Cancer Res.

[14] Winkel RR, von Euler-Chelpin M, Nielsen M, Petersen K, Lillholm M, Nielsen MB, Lynge E, Uldall WY, Vejborg I (2016). Mammographic density and structural features can individually and jointly contribute to breast cancer risk assessment in mammography screening: a case–control study. BMC Cancer..

[15] Kallenberg M, Petersen K, Nielsen M, Ng AY, Diao P, Igel C, Vachon CM, Holland K, Winkel RR, Karssemeijer N, Lillholm M (2016). Unsupervised deep learning applied to breast density segmentation and mammographic risk scoring. IEEE Trans Med Imaging.

[16] Malkov S, Shepherd JA, Scott CG, Tamimi RM, Ma L, Bertrand KA, Couch F, Jensen MR, Mahmoudzadeh AP, Fan B, Norman A, Brandt KR, Pankratz VS, Vachon CM, Kerlikowske K (2016). Mammographic texture and risk of breast cancer by tumor type and estrogen receptor status. Breast Cancer Res.

[17] Beckmann MW, Brucker C, Hanf V, Rauh C, Bani MR, Knob S, Petsch S, Schick S, Fasching PA, Hartmann A, Lux MP, Haberle L (2011). Quality assured health care in certified breast centers and improvement of the prognosis of breast cancer patients. Onkologie.

[18] Ursin G, Astrahan MA, Salane M, Parisky YR, Pearce JG, Daniels JR, Pike MC, Spicer DV (1998). The detection of changes in mammographic densities. Cancer Epidemiol Biomark Prev.

[19] Tibshirani R (1996). Regression shrinkage and selection via the lasso. J Roy Stat Soc: Ser B (Methodol).

[20] Friedman JH (2001). Greedy function approximation: a gradient boosting machine. Ann Stat.

[21] Bühlmann P, Hothorn T (2007). Boosting algorithms: regularization, prediction and model fitting. Stat Sci..

[22] Breiman L (2001). Random forests. Mach Learn..

[23] Pencina MJ, D’Agostino RB, Steyerberg EW (2011). Extensions of net reclassification improvement calculations to measure usefulness of new biomarkers. Stat Med.

[24] Bovelstad HM, Nygard S, Storvold HL, Aldrin M, Borgan O, Frigessi A, Lingjaerde OC (2007). Predicting survival from microarray data–a comparative study. Bioinformatics.

[25] Durning MV. Breast Density Notification Laws by state—interactive map. http://www.diagnosticimaging.com/breast-imaging/breast-density-notification-laws-state-interactive-map. Accessed 15 Nov 2015.

[26] Kerlikowske K, Zhu W, Tosteson AN, Sprague BL, Tice JA, Lehman CD, Miglioretti DL, Breast Cancer Surveillance C (2015). Identifying women with dense breasts at high risk for interval cancer: a cohort study. Ann Intern Med.

[27] Häberle L, Hein A, Rübner M, Schneider M, Ekici AB, Gass P, Hartmann A, Schulz-Wendtland R, Beckmann MW, Lo WY, Schroth W, Brauch H, Fasching PA, Wunderle M (2017). Predicting triple-negative breast cancer subtype using multiple single nucleotide polymorphisms for breast cancer risk and several variable selection methods. Geburtshilfe Frauenheilkd.

[28] Litjens G, Kooi T, Bejnordi BE, Setio AA, Ciompi F, Ghafoorian M, van der Laak JA, van Ginneken B, Sanchez CI. A survey on deep learning in medical image analysis. arXiv preprint arXiv:1702.05747. 2017. 10.1016/j.media.2017.07.005 28778026

[29] Wessels LF, Reinders MJ, Hart AA, Veenman CJ, Dai H, He YD, van’t Veer LJ (2005). A protocol for building and evaluating predictors of disease state based on microarray data. Bioinformatics.

[30] Varma S, Simon R (2006). Bias in error estimation when using cross-validation for model selection. BMC Bioinform.

[31] Schild RL, Maringa M, Siemer J, Meurer B, Hart N, Goecke TW, Schmid M, Hothorn T, Hansmann ME (2008). Weight estimation by three-dimensional ultrasound imaging in the small fetus. Ultrasound Obstet Gynecol.

